# Smooth predictions for age-period-cohort models: a comparison between splines and random process

**DOI:** 10.1186/s12874-025-02629-8

**Published:** 2025-07-28

**Authors:** Connor Gascoigne, Andrea Riebler, Theresa Smith

**Affiliations:** 1 MRC Centre for Environment and Health, Department of Epidemiology and Biostatistics, School of Medicine, Imperial College London, London, UK; 2 Department of Mathematical Sciences, Norwegian University of Science and Technology, Trondheim, Norway; 3 Department of Mathematical Sciences, University of Bath, Bath, UK

**Keywords:** Age-period-cohort, identifiability, smoothing, prediction, penalised splines, random processes, Bayesian model

## Abstract

**Background:**

Age-Period-Cohort (APC) models are well used in the context of modelling health and demographic data to produce smooth predictions of each time trend. When producing smooth predictions in the context of APC models, there are two main schools, frequentist using penalised splines, and Bayesian using random processes with little crossover between them.

**Methods:**

We compared prediction using APC models in either a frequentist or Bayesian paradigm using theory, simulated data, and two separate real-world data examples for mental ill-health outcomes. For the theoretical comparison, we describe each method and give an accessible description highlighting how the two methods are equivalent. For the simulated and real-world data, we compared the results for both in-sample (estimation) and out-of-sample (forecasting) prediction.

**Results:**

During the simulation study, the estimation results for both the penalised splines and random processes were almost identical. For the forecasting results, the random processes performed better. For the real-world examples, the estimation results for both were extremely close with random processes proving slightly better. For the real-world data forecasting results, the random processes provided a significant improvement over penalised splines.

**Conclusions:**

The combination of theory and data examples we presented here make the relationship between splines and random processes both accessible and interpretable. Whilst there is a theoretical link between both penalised splines and random processes, when forecasting is the goal, a Bayesian random process approach displayed better predictive properties in comparison to the frequentist penalised spline approach.

**Supplementary Information:**

The online version contains supplementary material available at 10.1186/s12874-025-02629-8.

## Introduction

Two important goals for any public health researcher interested in modelling how disease and demographic rates vary over time are (1) validating hypotheses about what is driving the underlying phenomena of interest and (2) forecasting the evolution of rates into the future. Both of these goals involve producing predictions: modelling is equivalent to in-sample predictions, and forecasting is equivalent to out-of-sample predictions. The production of smooth predictions that fulfil these goals are vital for data-driven approach to effective and efficient policy evaluation and recommendation.

The class of so-called Age-Period-Cohort (APC) models is a popular tool for modelling and predicting the evolution of rates over time when there are different patterns of change by age group. In APC models, we consider three-time scales: age—the age of an individual when the event of interest occurs; period—the time (often a year) that the event occurs; and cohort—the time (usually a year) that the individual was born. Applications of APC models can be found in a range of public health contexts, such as modelling prostate cancer, thyroid cancer, stomach cancer, and obesity [1–4], and sociological contexts, such as exploring trends in suicide or opioid deaths [5, 6].

Although popular, users of APC models must overcome a key technical issue called the ‘APC identification problem.’ Because the three-time scales of interest are intrinsically connected (e.g., given the birth year (cohort) and age at the event of an individual, we can calculate the year of the event (period) as cohort + age), we cannot estimate linear trends along all three-time scales simultaneously. A common solution to the identification problem is to parameterise the temporal trends into identifiable quantities that can be fully estimated.

On top of the identification problem, additional identification issues may arise (in the form of artificial cyclic patterns in the estimates) when the groups for age, period, and cohort are aggregated in non-equal interval widths (i.e., groups that do not contain the same number of years). In these scenarios, it has been shown that smoothing in APC models is an effective method to relieve the additional identification problems [7, 8]. However, Gascoigne and Smith [9] have shown that the effectiveness of the smoothing function is dependent upon its specification, and for estimates that alleviate the additional identification problem regardless of the parameterisation, a penalty on the second derivative (a measure of how ’wiggly’ the function is) is essential. For smoothing in a frequentist setting, splines are often used, while random processes (i.e., random walk models) are used in a Bayesian setting. For smoothing with a penalty on the second derivative, (cubic) penalised smoothing splines and random walk of second order (RW2) random processes are standard approaches used in frequentist and Bayesian settings, respectively.

Forecasting, or predicting, is important for policy planning tasks such as the allocation of public health funds. When predicting, there is often no single best temporal scale to use for prediction, making the APC model well placed for the task. However, the APC model is generally less accepted often due to the associated identification problem [10]. When an APC model is formulated appropriately, predictions are not affected by the identification problem [11] and have frequently been used in a Bayesian paradigm [12–16]. Forecasts using APC models are commonly used in the context of cancer incidence [17, 18].

With a fully identifiable model, parameter estimation can be performed in a frequentist or Bayesian setting. In this article, we compare common model specifications for both inference approaches based on in-sample (estimation) and out-of-sample (forecasting) predictions to highlight important differences in how sources of uncertainty are captured.

The rest of the article is as follows. In the “Self-harm and alcohol related deaths in England and Wales, 2006-2021” section, we present the alcohol and self-harm deaths data used as a real data illustration. In the “Age-period-cohort model” section, we present the APC model. In the “Smoothing approaches” section, we give an explanation of smoothing approaches using penalised splines and random processes and highlight the theoretical link between them. In the “Implementation” section, we discuss our implementation of spline and random process models. The “Comparing predictive capabilities” section provides two data examples (simulated and real-world) comparing the smooth predictions of the spline and random process models for each. Finally, in the “Discussion” section, we finish with a discussion.

## Self-harm and alcohol related deaths in England and Wales, 2006-2021

The World Health Organization (WHO) has identified increasing mental health awareness as a key target to achieve its Sustainable Development Goal 3.4 [19], due to significant associations between mental health disorders and non-communicable diseases [20]. With over 700,000 global deaths from suicide each year, suicide is the fourth leading cause of death among those aged 15–29 [21] and is a key indicator of mental illness. In the United Kingdom (UK), there are, on average, 6,311 suicide deaths annually. Whilst this has declined in recent years [22], a single suicide is estimated to cost the UK economy £1.46 million [23]. Specifically, within suicide-related deaths, alcohol abuse—which has a strong co-morbidity with mental illnesses such as anxiety and depression [24]—is a significant factor, with an average of 542 alcohol misuse-specific deaths in the UK annually [25]. With the ever-increasing mental health [26] and alcohol misuse crisis [27] in the UK, a wide range of methods is essential to fully explore, understand, and predict future trends in a public health context, ensuring that appropriate measures can be implemented. APC models are often used to analyse suicide rates by examining differences due to age, short-term (period), and long-term (cohort) factors. Smoothing functions for APC models have been applied to study suicides in Brazil [28], China [29], Hong Kong and Taiwan [30], Korea [31], and Switzerland [32], among others.

In this article, we use mortality attributable to suicide in England and Wales to compare frequentist and Bayesian smoothing methods in the context of APC models. From the UK’s Office for National Statistics (ONS), we downloaded counts of suicide using the International Classification of Diseases version 10 (ICD-10) codes F10 (mental and behavioural disorders due to use of alcohol) and X60-X84 (intentional self-harm). The counts were given in yearly periods from 2006 to 2021 and in five-year age bands between the ages of 25 to 85. The data from 2013 onwards was extracted through the ONS Nomis tool (https://www.nomisweb.co.uk/) and data from 2006 to 2012 was assembled from individual annual reports. In addition, mid-year population estimates were extracted by five-year age groups in all years from the ONS Nomis tool. For the ICD 10 codes F10 and X60 – X84, there are, on average, 46.63 and 299.13 events for age-year combination, respectively. Given the two different magnitudes of observations, we explore whether the magnitude of the observation has an effect on the performance of either the frequentist or Bayesian smoothing methods.

In the following, we refer to these as alcohol (ICD 10 code: F10) and self-harm (ICD 10 codes: X60-X84) related deaths.

**Fig. 1:**
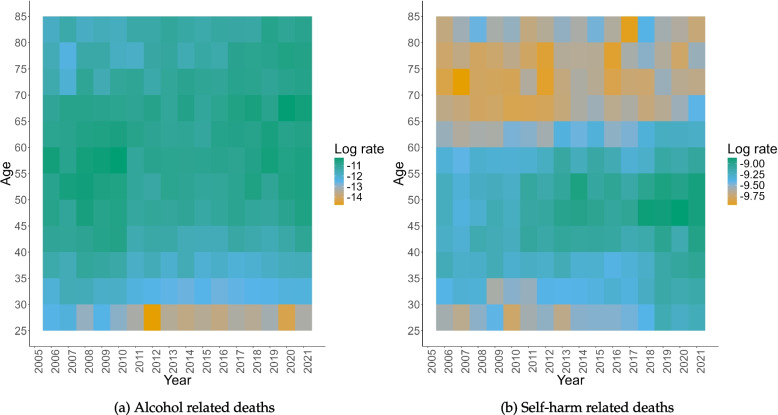
Deaths due to alcohol (**a**) and self-harm (**b**) for the years 2006–2021 and ages 24–84. Period is grouped into single years and operates along the *x*-axis. Age is grouped into five-year ages groups and operates along the *y*-axis. Cohort operates along the $y = x$ axis. Deaths are reported as log-rates with the dark-to-light colouring indicating lower-to-higher rates

Figure 1 shows the age-specific log-rates over time for both alcohol- and self-harm-related deaths. Using the natural logarithmic scale allows for a closer inspection of the trends in age, period, and cohort. An extra 1/2 event was added to each mortality count to avoid taking logs of 0. For both causes, there is substantial variation in risk according to age (looking along the *y*-axis). For example, the risk of alcohol-related deaths is much lower for the younger age groups (25-30 and 30-35) than in any other age group, and the risk of death related to self-harm is lowest in the youngest (25-30) and the older (60+) age groups, with the ages in between having much higher rates.

There is some evidence of trends over the year of death, but they are small in comparison to trends in age of death. Finally, there are also age-by-year interactions that could indicate cohort effects. For example, ages 70+ had a lower risk of alcohol-related deaths in the years 2006-2010 than in more recent years, and ages 25-30 had a lower risk of death relating to self-harm in the years 2006-2013 than in 2013 and onwards.

## Age-period-cohort model

Let $y_{ap}$ be our response variable of interest, which can be modelled via any distribution from the exponential family of distributions (i.e., Poisson, negative Binomial, binomial, Gaussian, gamma, exponential, Weibull) for age group *a* and period *p* where $a = 1, \dots , I$ and $p = 1, \dots , J$. The cohort index varies $c = 1, \dots , K = M \times \left( {I - a}\right) + p$ with *M* as the ratio between the number of years in the age group to the number of years in the period group. A general APC model for the mean function of the distribution can be written as,

1 $$\begin{aligned} d\left( {\mu _{ap}}\right) = \eta _{ap} = \beta _0 + h_{A}\left( {a}\right) + h_{P} \left( {p}\right) + h_{C}\left( {c}\right) . \end{aligned}$$

Here $d\left( {\cdot }\right)$ is the link function for the appropriate distribution (e.g., the logit for the binomial distribution), $\mu _{ap} \equiv \mathbb {E}\left[ {y_{ap}}\right]$ is the expected value of the response, $\eta _{ap}$ is the linear predictor, $\beta _0$ is an intercept, and $h_{A}$, $h_{P}$, and $h_{C}$ are functions of age, period, and cohort. The linear components of these three temporal functions in this model cannot be simultaneously estimated due to the identification problem (cohort = period - age). A common approach to resolve the identification issue is to reparametrise into identifiable quantities. One such method was proposed by Holford [33], where the temporal terms were partitioned into a linear trend (slope) and their respective (orthogonal) functions of curvature, with the latter being identifiable. Following Holford [33], Eq. 1 can be written as the following identifiable APC model,

2 $$\begin{aligned} \eta _{ap} = \beta _0 + \beta _1 a + \beta _2 p + f_{A}\left( {a}\right) + f_{P}\left( {p}\right) + f_{C}\left( {c}\right) . \end{aligned}$$

In Eq. 2, $\beta _1$ and $\beta _2$ are slopes for age and period, respectively and $f_{A}, f_{P},$ and $f_{C}$ are the age, period, and cohort functions of curvature.

In any reparameterisation of an APC model, an arbitrary choice has to be made. In Eq. 2, that choice is which two (out of the three) temporal slopes to include. Holford [33] showed that the choice of slope to drop does not affect the curvature estimates or predictions (in- and out-of-sample). However, one must be cautious not to over interpret estimates of the remaining two slopes, which capture some aspect of the linear trend in the third time scale. In this work, we make the arbitrary choice to drop the cohort slope, which means $\beta _1$ and $\beta _2$ are the linear trends in age + cohort and period + cohort, respectively. The interpretation of the linear trends is analogous if the age or period slope is dropped instead.

For the rest of this article, we will focus on the specification of $f_A$, $f_P$ and $f_C$. However, there are many other choices a data analyst must make such as selecting the distribution for $y_{ap}$ and whether a simpler sub-model, such as an age-period model, may be more appropriate than the full age-period-cohort model. Whilst we will not touch on these choices in this article, it is worth noting that the frequentist and Bayesian paradigms have quite different approaches to model development and comparison.

For the data described in the “Self-harm and alcohol related deaths in England and Wales, 2006-2021” section, we have $I = 12$ age groups, $J = 16$ periods, and the data is grouped into unequal intervals with the period being in a single year and age being in five-year interval widths (i.e., $M = 5$ in the cohort index). When APC models are fit to data aggregated in such a way, the (previously identifiable) curvature terms of the period and cohort are no longer identifiable and display an artificial cyclic pattern [7, 8, 34]. Modelling the curvature functions using smoothing functions has been used as an effective solution to this additional form of identifiability issues, but this depends upon arbitrary choices in how to define the choice of smoothing function. It has been shown the estimates can be robust to both the additional identification problem and the specification of the smoothing function when including a penalty term on the second derivative of the estimate of the smoothing function [9].

## Smoothing approaches

We now describe the theoretical parallels between the smoothing approaches of penalised splines and random processes. The connection has been discussed previously in the context of ecology and the use of stochastic partial differential equations [35]. However, we consider the connection in relation to health and demographic modelling and are using random walk models within the context of an APC framework.

For the purpose of explanation, we describe smoothing for a simple univariate function and consider the simple one-dimensional smooth over age:

$$\begin{aligned} \eta _{ap} = \beta _0 + \beta _1 a + f_{A}\left( {a}\right) . \end{aligned}$$

### Penalised regression splines

In a frequentist paradigm, an estimator of $f_{A}$ can be found by maximising the following penalised log-likelihood,

3 $$\begin{aligned} \widehat{\beta }, \widehat{f_{A}}, \widehat{\theta } = \underset{\beta , f_{A}, \theta }{\text {arg}\,\text {max}} \left[ l\left( {\varvec{\beta }, \varvec{f}_{A}, \varvec{\theta }}\right) + \lambda \int f_{A}^{\prime \prime }\left( {a}\right) ^2 d{a}\right] , \end{aligned}$$

where $l\left( {\varvec{\beta }, \varvec{f}_{A}, \varvec{\theta }}\right) = \log \mathcal {L}\left( {\varvec{\beta }, \varvec{f}_{A},\varvec{\theta }}\right)$ is the log-likelihood, and $\lambda \int f_{A}^{\prime \prime }\left( {a}\right) ^2 d{a}$ is a penalty function on the second derivative of the smooth function $f_{A}$ with smoothing parameter $\lambda$ that controls the trade-off between model fit and smoothness. The inclusion of the penalty function on the second derivative of $f_{A}$ penalises $f_{A}$ when it deviates from linearity. If $\lambda = 0$, there is no cost for fitting complicated functions, and $\widehat{f_{A}}$ can be extremely ‘wiggly’. As $\lambda \rightarrow \infty$, the cost for fitting a complicated function increases and $\widehat{f_{A}}$ is forced to be closer to a simple polynomial.

To make maximising Eq. 3 tractable, we use a finite basis approximation to true function $f_{A}$. Within the context of APC modelling, splines have been used to approximate the true function $f_{A}$ on several occasions [7, 36–39]. A spline basis is a set of polynomial (basis) functions which are based on points called knots. Given $g_t\left( {x}\right)$, the $t^\text {th}$ basis function, *f* is approximated with a spline as follows,

$$\begin{aligned} f_{A}\left( {a}\right) = \sum \limits _{t = 1}^{T} g_{t}\left( {a}\right) \gamma _{t} = \varvec{Z}\varvec{\gamma }, \end{aligned}$$

where *T* is the number of basis functions, $\gamma _{t}$ are the unknown weights to be estimated and $\varvec{Z}$ is an $n \times T$ matrix of basis vectors. The classical spline literature distinguishes between ‘smoothing splines’, where the number basis vectors *T* is tied to the number of observed data locations (e.g., the *I* age groups) and ‘regression splines’, where the number of basis vectors is substantially less than the data input dimension [40]. While we focus on the latter case in this article, we note that in the APC literature the distinctions between the ’smoothing’ and ’regression’ terminology is less strict. For example, [7] uses the term ‘smoothing splines’ when in fact ‘regression splines’ would be more precise.

The functions $g_t(\cdot )$ are typically simple curves defined relative to pre-specified knots. We consider three popular choices of spline basis functions: Thin Plate Regression Splines (TPRS), Cubic Regression Splines (CRS) and B-Splines (BS). Whilst all are suitable for our application, they have their advantages and disadvantages. TPRS can smooth in multiple dimensions and do not necessarily need the number of knots specified, but they are computationally expensive. CRS are computationally cheaper than TPRS, but only smooth in one dimension. BS are sparse and can be flexibly paired with penalties of different orders, however this causes the interpretation of the penalty to be less clear when compared to derivative-based methods. Figure S1 in the Supplementary Material shows examples of a CRS, BS and TPRS bases defined by five knots. For a detailed description of these spline bases and others, see ([41], Chapter 5).

With a given basis representation, $\varvec{g}$, with unknown weights, $\varvec{\gamma }$, the penalty function for $f_{A}$ can be rewritten,

$$\begin{aligned} \int f_{A}^{\prime \prime }\left( {a}\right) ^2 d{a} = \varvec{\gamma } \int \varvec{g}^T\left( {a}\right) \varvec{g}\left( {a}\right) d{a} \varvec{\gamma } = \varvec{\gamma } \varvec{S} \varvec{\gamma }, \end{aligned}$$

where $\varvec{S} = \int \varvec{g}^T\left( {a}\right) \varvec{g}\left( {a}\right) d{a}$ is known as the penalty matrix. Therefore, the penalised log-likelihood to be maximised can be re-written in terms of the finite basis approximation to each of the smooth functions,

4 $$\begin{aligned} l_p\left( {\varvec{\beta }, \varvec{\gamma }, \varvec{\theta }}\right) = l\left( {\varvec{\beta }, \varvec{\gamma }, \varvec{\theta }}\right) + \lambda \varvec{\gamma } \varvec{S} \varvec{\gamma }. \end{aligned}$$

where $l_p\left( {\varvec{\beta }, \varvec{\gamma }, \varvec{\theta }}\right) = \log \mathcal {L}_p\left( {\varvec{\beta }, \varvec{\gamma }, \varvec{\theta }}\right)$ is the penalised log-likelihood that is maximised by $\widehat{\varvec{\beta }}$, $\widehat{\varvec{\gamma }}$ and $\widehat{\varvec{\theta }}$, where $\varvec{\theta }$ is a vector of any other parameters in the likelihood (e.g., the dispersion parameter in a negative binomial model).

**Fig. 2:**
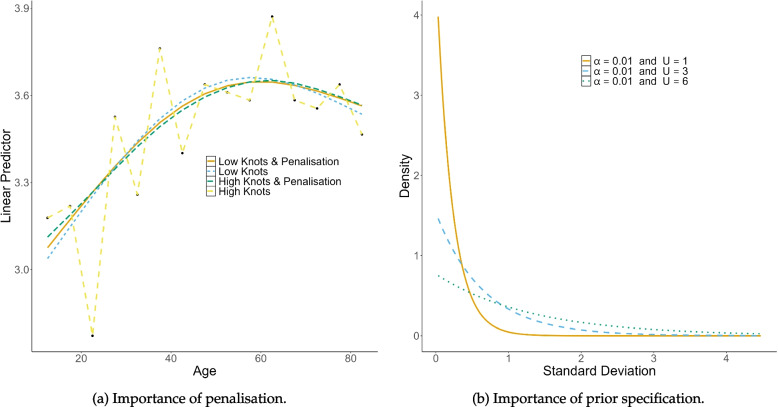
The left-hand plot (**a**) shows the importance of penalisation (see the “Penalised regression splines” section) to reducing overfitting. Four thin plate regression spline models with or without penalty and with large or small number of knots are fit to toy data (black dots). The right-hand plot (**b**) shows the importance of prior specification (see the “Connections between penalised regression splines and random processes” section). Three different PC prior specifications of the standard deviation $\sigma = \sqrt{1/\tau }$ are compared for different choices of *U* in $\text {P}(\sigma>U) = \alpha$, with $\alpha =0.01$

Where pre-specification of knots is required, an important aspect is selecting the number of knots to use and where to place them. The more knots, the more flexible the spline is, but without a penalty, this can lead to overfitting. Using a toy dataset, Fig. 2a highlights how a penalty reduces the importance of the selection of the number of knots. In the toy dataset, the number of unique ages was $A = 15$. We used 3 and 15 knots to define the low and high knot choices, respectively. High knots without a penalty clearly overfit. High knots with a penalty form a smooth curve similar to that of low knots. The inclusion of the penalty ensures there is no overfitting, while maximising the amount of information to be gained by including more knots.

### Random processes

In the Bayesian paradigm, random processes are commonly used for smoothing by specifying a multivariate distribution for the function of interest (e.g., $f_A$) evaluated at a finite number of points (e.g., $a = 1, \dots , I$). For temporal smoothing in public health and sociological contexts, random walks of order 1 and 2 (abbreviated RW1 and RW2, respectively) are extremely popular. They are widely used within APC models for both estimation and prediction [8, 12–14, 42–45]. Recently, Okui [46] used random walk priors in an APC model to analyse the prevalence of common psychiatric disorders in Japan. RW1 and RW2 models achieve smoothing by penalising deviations from a constant or linear trend, respectively, and as they are (intrinsic) Gaussian Markov random fields (GMRFs; [47]) with sparse inverse covariance matrices, i.e., precision matrices, they offer good computational properties.

Similarly to the penalised regression splines, a RW2 penalises deviations in linearity [47]. Assuming $\varvec{f}_{A}$ follows a RW2 model, the second differences have the distribution,

$$\begin{aligned} \Delta ^2 {f_{A}}(a) \overset{\text {iid}}{\sim } \text {N}\left( {0, \tau ^{-1}}\right) ,\, a < I-2, \end{aligned}$$

where there is a flat prior on first two time points and $\tau$ is the precision (inverse variance) parameter. The precision $\tau$ controls the trade-off between smoothness and closeness to the data (it is the smoothing parameter) and has parallels with the smoothing parameter $\lambda$ in the spline framework. For example, as $\tau \rightarrow \infty$, the distribution of $f_{A}$ shrinks towards a straight line. The joint density of $\varvec{f}_{A}$ is defined,

5 $$\begin{aligned} & \pi \left( {\varvec{f}_{A} | \tau }\right) \propto \tau ^{{\left( {I - 2}\right) }/{2}} \exp \nonumber \\ & \left( {-\frac{\tau }{2} \sum \limits _{a = 1}^{I - 2} \left[ { {f_{A}}(a) - 2{f_{A}}(a+1) + {f_{A}}(a+2) }\right] ^2 }\right) \nonumber \\ & \qquad \qquad \quad = \tau ^{{\left( {I - 2}\right) }/{2}} \exp \left( { \frac{1}{2} \varvec{f}_{A}^T \varvec{Q} \varvec{f}_{A} }\right) , \end{aligned}$$

with precision matrix

6 $$\begin{aligned} \varvec{Q} = \tau \varvec{R} = \tau \left( \begin{array}{ccccccc} 1 & -2 & 1 & & & & \\ -2 & 5 & -4 & 1 & & & \\ 1 & -4 & 6 & -4 & 1 & & \\ & \ddots & \ddots & \ddots & \ddots & \ddots & \\ & & 1 & -4 & 6 & -4 & 1 \\ & & & 1 & -4 & 5 & -2 \\ & & & & 1 & -2 & 1 \\ \end{array}\right) , \end{aligned}$$

where $\varvec{R}$ is referred to as the structure matrix of rank $I-2$. Due to this, the RW2 precision matrix, $\varvec{Q} = \tau \varvec{R}$, is rank deficient, so that the RW2 is an improper or intrinsic GMRF. Of note, the definition of the RW1 is based on the first differences and analogous to the definition of the RW2, for details we refer to Rue and Held (Section 3.3.1; [47]).

### Connections between penalised regression splines and random processes

There are theoretical parallels between estimates produced from penalised regression spline models and random walk models based on theory from functional analysis. We wish to identify the key information from this theory so that a general practitioner can make the connection and use the methods interchangeably. When estimating $f_{A}$, we have shown above that we can either define a finite-dimensional approximation to $f_{A}$ and then estimate the parameters of the approximation by maximising the penalised log-likelihood, or we can place a prior on $f_{A}$ and define estimates of the model parameters by evaluating the posterior distribution via Bayes rule.

To see the relation between a penalised regression spline and RW2 prior model, we rewrite the penalised log-likelihood in Eq. (4) in terms of a likelihood,

$$\begin{aligned} \mathcal {L}_p\left( {\varvec{\beta }, \varvec{\gamma }, \varvec{\theta }}\right) = \mathcal {L}\left( {\varvec{\beta }, \varvec{\gamma }, \varvec{\theta }}\right) \times \exp \left( {- \lambda \varvec{\gamma }^T \varvec{S} \varvec{\gamma }}\right) . \end{aligned}$$

If we consider the key concept in Bayesian inference ($posterior \propto prior \times likelihood$), $\mathcal {L}_p\left( {\varvec{\beta }, \varvec{\gamma }, \varvec{\theta }}\right)$ and $\mathcal {L}\left( {\varvec{\beta }, \varvec{\gamma }, \varvec{\theta }}\right)$ are equivalent to the posterior distribution and the likelihood function, respectively and $\exp \left( {- \lambda \varvec{\gamma }^T \varvec{S} \varvec{\gamma }}\right)$ can be thought of as the prior distribution of the parameters $\varvec{\gamma }$. The prior distribution can be written,

$$\begin{aligned} p\left( {\varvec{\gamma } | \lambda }\right) \propto \exp \left( {- \varvec{\gamma }^T \varvec{Q}_\lambda \varvec{\gamma }}\right) , \end{aligned}$$

where $\varvec{Q}_\lambda = \lambda \varvec{S}$ is of the form of an improper GRF prior on $\varvec{\gamma }$ with mean zero and precision $\varvec{Q}_\lambda$, i.e., $\varvec{\gamma } \sim \text {N}\left( {\varvec{0}, \varvec{Q}_\lambda ^{-1}}\right)$, [41, 48]. The GRF is improper as it is rank deficient by the size of the null space of the penalty matrix, $\varvec{S}$. In our penalised spline, the dimension of the null space is two, therefore the precision matrix is of an improper GRF with rank $I-2$. Thus, a penalised spline can be seen from a Bayesian view as placing an improper, zero-mean GRF prior with rank $I-2$ precision matrix on $\varvec{\gamma }$. The RW2 model is an example of one such prior, and furthermore, for some spline representations, $\varvec{S}$ can have exactly the same tri-diagonal form as $\varvec{R}$ in Eq. 6 [41].

Whilst both the penalised regression spline model and the RW2 prior model are imposing a penalty on the second derivative of the estimates, how each model performs this differs which causes slight differences in practical estimates. For example, the smoothing parameter $\lambda$ of the penalised regression spline model can be estimated by cross validation [41], whereas in the RW2 prior model, it is common to specify a distribution for the smoothing parameter $\tau$, *a priori* and consequently determine its posterior marginal distribution within a Bayesian inference framework [47]. As recommended by Simpson et al. [49] we use penalised complexity (PC) prior for $\tau$, which is a type-2 Gumbel distribution for $\tau$ or equivalently an exponential distribution on the standard deviation $\sigma = \sqrt{1/\tau }$ with parameter $\kappa$. The rate parameter $\kappa$ is chosen based on a probability contrast for $\sigma$, specifying $\text {Prob}(\sigma>U) = \alpha$, with $U>0$ and $\alpha \in (0,1)$, such that $\kappa = -\ln (\alpha )/U$. For details, we refer to Simpson et al. [49]. It is worth noting that penalised regression splines can also be fit in a fully Bayesian workflow, (c.f, Ruppert et al. [50], Bauer et al. [51]); however, we have not included a Bayesian implementation of splines in our comparisons because, to the best of our knowledge, this has not been considered in an APC context.

## Implementation

While there are many programming languages capable of fitting the models described in our manuscript, we have chosen to use R (version 4.2.2; [52]) due to its extensive statistical libraries, active community support, and suitability for reproducible research. The choice of R reflects the authors expertise and the broad adoption of R in the statistical modelling community. There are several specialised software packages for APC analysis and prediction within both frequentist and Bayesian frameworks, such as the R-packages Epi, BAMP or BAPC but also many others. The R-package Epi [53], for example, implements the APC model in a frequentist setting using penalised splines as described in Carstensen [37]. The R-packages BAMP [54] and BAPC [16] use Bayesian inference. BAMP uses Markov chain Monte Carlo (MCMC) and assumes binomially distributed data. It can be used for both model estimation and prediction. The R-package BAPC focuses on prediction and uses Integrated Nested Laplace Approximation (INLA) for deterministic Bayesian inference. Both BAMP and BAPC use random walks of first or second order for the temporal random effects. In this work we chose to not use existing implementations, but to implement the APC model as parameterised in Eq. (2) from scratch to have full control over all parameters and the way inference is performed.

We fitted the penalised spline model with the Generalised Additive Model (GAM) framework, as implemented in the mgcv package [41]. This package offers a wide range of spline bases to represent smooth functions and their penalties. In mgcv, the syntax to fit a penalised spline on the term x is s(x, k, bs, fx = FALSE). The argument *k* is for the number of knots used to define the basis function and bs defines the type of basis function. For the CRS, BS and, TPRS functions, bs = ‘cr’, ‘bs’ and ‘tp’, respectively. The argument fx = FALSE (which is the default option) ensures a penalised (as opposed to un-penalised) spline is being fit.

We fitted the RW2 model within a Bayesian hierarchical framework. Here, several Bayesian inference engines could be used such as BUGS, Stan, or INLA. Given the APC model specified in Eq. 2 has observations are from any of the exponential family of distributions and the linear predictor is an additive combination of linear and non-linear terms which can be represented by a Gaussian process, it falls within the class of latent Gaussian models and can be modelled via INLA with the r-inla package [55], see www.r-inla.org. INLA provides accurate approximations of the marginal posterior distribution for all model parameters whilst avoiding the need for costly and time-consuming MCMC sampling. In r-inla, the syntax to fit a RW2 prior on the term x is f(x, model = ‘rw2’, hyper, ...). The argument model = ‘rw2’ specifies we fitted a RW2 model. By fitting a RW2 model r-inla will implicitly set the arguments contr = TRUE, rankdef = 2 to constrain the model which, in the case of a RW2, is to constraint against an intercept and linear trend which automatically forces the rank deficiency of the model to be two. The argument hyper is where the hyper priors are specified. In this work, we use PC priors with parameters $\alpha =0.01$ and either $U=1$, $U=3$ or $U = 6$, see Fig. 2b. Those are specified by setting list(prec = list(prior = ‘pc.prec’, param = c(*U*, $\alpha$))) appropriately.

## Comparing predictive capabilities

We compared predictions between the splines and random process under two different scenarios: simulated and real-world data. The real-world data was described in the “Self-harm and alcohol related deaths in England and Wales, 2006-2021” section and the simulated data is motivated by the alcohol-related deaths from the “Self-harm and alcohol related deaths in England and Wales, 2006-2021” section.

Forecasting plays a vital role in decision making by providing a data-driven approach to anticipate future outcomes. These can be used by policy makers to guide intervention policy and resource allocation by predicting how a disease progresses over time. Moreover, exploring differences in the forecasted values are used to identify profiles or sub-populations more at risk than others. This is vital for reducing inequalities. In the setting of APC modelling, smoothed predictions are often used to explore the effects of combinations of the three temporal trends (i.e., age-by-period or age-by-cohort) [56, 57].

The APC model in Eq. 2 is written in general and is suitable for any observation data that falls within the exponential family distribution (e.g., Poisson, negative binomial, binomial, normal, etc...). Furthermore, the APC identification problem occurs regardless of the distribution being considered. For the data described in the “Self-harm and alcohol related deaths in England and Wales, 2006-2021” section, we assume the observations are from a Poisson distribution and use a log-link function.

First we defined the index for age, period, and cohort when forecasting $t> 1$ periods ahead. Let $a = 1, \dots , I$ age groups. For estimation we used the periods $p = 1, \dots , J$ and the cohorts $c = 1, \dots , K = M \times \left( {I - a}\right)$. We made forecasts, using the periods $J + 1, \dots , J + t$, for the same group of ages, and cohorts $c = 1, \dots , K = M \times \left( {I - a}\right) + \left( {J + t}\right)$. We note that for some age groups the cohort effect does not need to be predicted as the cohort index repeats over several period indices [58].

The theoretical approach to forecasting was as follows. Under the spline model fit via mgcv, forecasts were made by defining the basis functions of the spline for the unobserved periods and then multiplying these by the estimated model regression coefficients. Under the random process model fit via r-inla, the posterior distribution of the conditional period effect was defined by the previous two time periods and a cubically increasing variance (Section 3.4.1; [47]). For both the spline and random process model, the method we used to define the cohort effect forecasts was analogous. Moreover, the forecasts of new observations were made by defining the linear predictor (Eq. 2) for each temporal effect using the unobserved combinations of age, period and cohort.

The practical approach to forecasting was as follows. Under the spline model fit via mgcv, the model was first fit using a dataset where there are only the observations to be estimated. The results of this model fit were then used in the mgcv::predict.gam function along with a new dataset of the unobserved combinations of age, period and cohort. Under the random process model fit via r-inla, a dataset containing all combinations (i.e., those used for estimation and those used for forecasting) of age, period and cohort was defined where the forecasted observations were pseudo-observations of NA.

We evaluated the predictive performance by evaluating both the point predictions and the predictive distribution. To assess point predictions, we used the Mean Absolute Error (MAE), and Mean Square Error (MSE) defined as

$$\begin{aligned} \text {MAE}_{ap} & = \frac{1}{N} \sum \limits _{n=1}^{N} |{\widehat{\eta }_{ap} - \eta _{ap}}|\quad \text {and}\\ \text {MSE}_{ap} & = \frac{1}{N} \sum \limits _{n=1}^{N} \left( {\widehat{\eta }_{ap} - \eta _{ap}}\right) ^2, \end{aligned}$$

where $n = 1, \dots , N$ are the number of simulations, and for age *a* and period *p*, $\widehat{\eta }_{ap}$ and $\eta _{ap}$ are the predicted and observed linear predictors, respectively.

We assessed the entire predictive distribution using the 95% Interval Score (IS) [59]. The IS is a scoring rule that transforms interval width and (empirical) coverage into a single score. Consider the estimate log rate $\widehat{\eta }_{ap}$ for each age *a* and period *p* combination is associated with lower and upper uncertainty, $\left[ {l_{ap}, u_{ap}}\right]$, defined using the respective $\left( {1 - \alpha }\right) \cdot 100 \%$ lower and upper predictive quantiles. The IS for $\alpha \in \left( {0,1}\right)$ is defined,

$$\begin{aligned} \text {IS}_{\alpha }\left( {\eta _{ap}}\right) & = \left( {u_{ap} - l_{ap}}\right) + \frac{2}{\alpha }\left( {l_{ap} - \eta _{ap}}\right) \mathbb {I}\left[ {\eta _{ap} < l_{ap}}\right] \\ & \quad + \frac{2}{\alpha }\left( {\eta _{ap} - u_{ap}}\right) \mathbb {I}\left[ {\eta _{ap}> u_{ap}}\right] , \end{aligned}$$

where $\mathbb {I}\left[ {\cdot }\right]$ is an indicator function that penalises how many data points, here $\eta _{ap}$, are outside the interval. The final IS score was defined by averaging over all IS’s, $\text {IS}_{\alpha } = \Sigma _{ap} \text {IS}_{\alpha }\left( {\eta _{ap}}\right)$. A lower IS is indicative of a better performing model.

For the MAE, MSE and IS, we defined point predictions and their associated uncertainty for both the penalised spline and RW2 models. For the non-Bayesian penalised spline model fit via mgcv, point predictions were defined by maximising the penalised likelihood. The associated uncertainty was calculated by adding and subtracting the standard error for each estimate multiplied by 1.96, the 97.5th percentile point of the normal distribution. The standard errors in mgcv are based on the Bayesian posterior covariance matrix (Section 6; [41]). For Bayesian RW2 model fit via r-inla, point predictions were defined as the posterior median, i.e., 50% quantile of the posterior marginal distribution of interest (i.e., the linear predictor). The associated uncertainty was defined as the 2.5% and 97.5% posterior quantiles.

### Using simulated data

We first compared predictions using simulated data. The simulation study is motivated by the alcohol-related deaths from the “Self-harm and alcohol related deaths in England and Wales, 2006-2021” section. The shapes for the age, period, and cohort effects are adopted from a simulation study for Gaussian data from Luo and Hodges [60]. To keep the shapes of the age, period, and cohort functions but make the responses representative of the rare alcohol-related deaths example, we included a scale and shift alongside the functions of Luo and Hodges [60]. A similar alteration was performed in Gascoigne and Smith [9].

We generated mortality counts using single-year ages (from 10 to 84) and periods (2000 to 2020). We fixed 750,000 as the population at risk for each age-period combination to align with the population size in the alcohol and self-harm-related deaths example. To mimic the reality of data collection and dissemination, we generated data in single-year age-period combinations and then aggregated age into five-year groups, i.e., $10 - 14, 15 - 19, \dots , 80 - 84$. When modelling, we used the midpoint of the age groups, i.e., $12.5, 17.5, \dots , 82.5$. We simulated $n = 1, \dots , N = 100$ data sets in this way. For each data set, we assessed both the estimation and forecasting capabilities. To fit the model, we used the data for the years 2000 to 2017, thus we used this year range for estimation. Using the model fit to the 2000 to 2017 data, we produced forecasts for the years 2018 to 2020 and compared this to the simulated data.

For the simulation study, we used three common spline basis functions discussed previously: CRS, BS, TPRS. As shown in Fig. 2a, the choice of the number of knots is less important when including a penalty. Consequently, we use 10, 10, and 12 for the number of age, period, and cohort knots, respectively, when defining their basis functions. We fit a RW2 model with three different PC prior specifications. For all specifications, we used $\alpha = 0.01$ and either $U = 1$, $U = 3$, or $U = 6$. In the Manuscript, we present the results of the simulation study where we modelled the mortality counts using a Poisson likelihood with a log-link function and included a population offset. In the Supplementary Material, we include the results of the simulation study using a negative Binomial likelihood. This was conducted as a sensitivity analysis, the results of which are consistent with the results of the Poisson likelihood presented here.

**Fig. 3:**
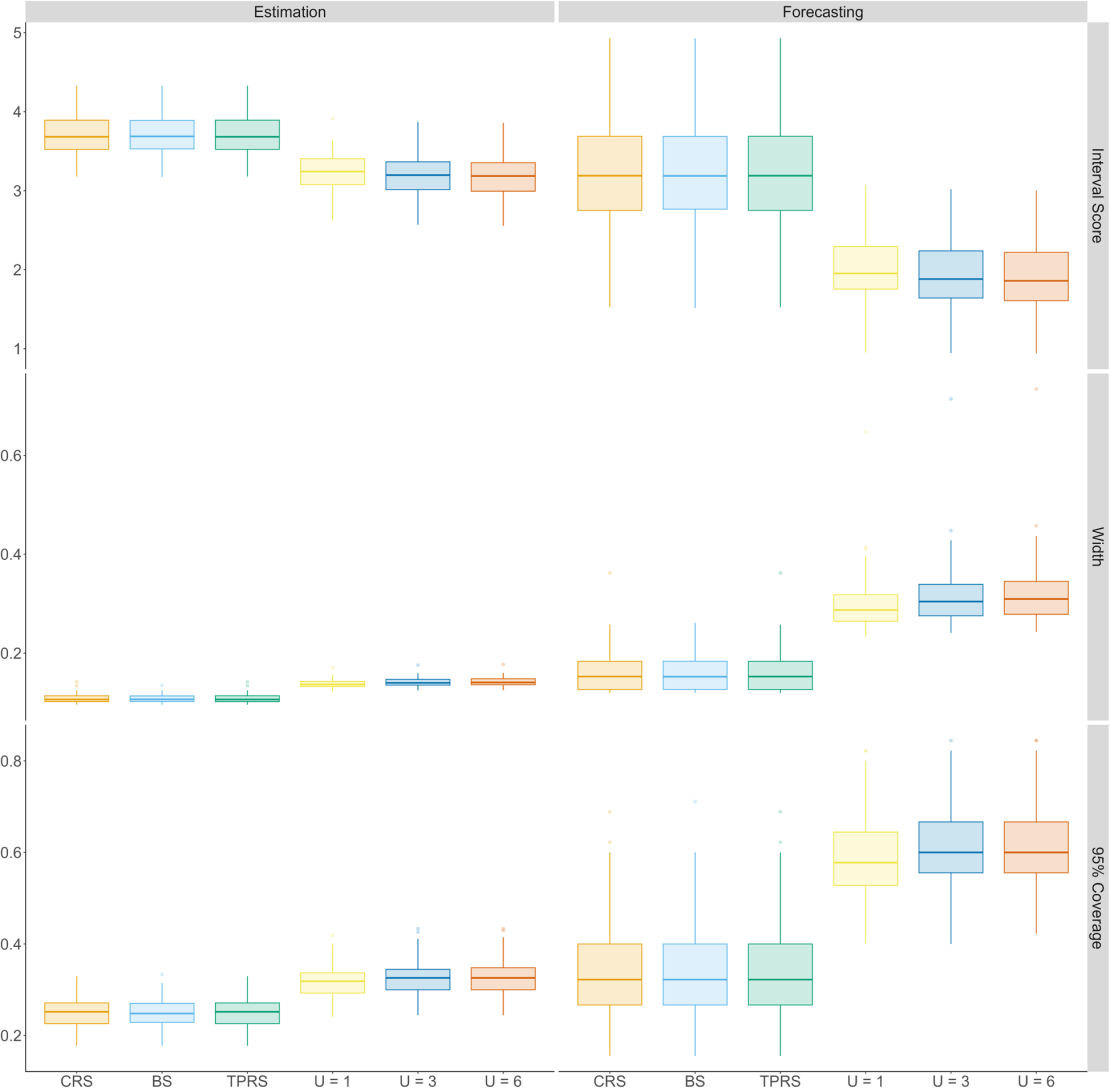
Boxplots for the interval score, (uncertainty) width, and (empirical) coverage for the simulation study. The column facet are the scores for the estimated (left) and forecasted (right) values, respectively. The row facets are for each of the scores listed above going from top-to-bottom, respectively. In each facet, the first three boxes are spline models defined with Cubic Regression Splines (CRS), B-Spline (BS) and Thin Plate Regression Spline (TPRS) basis. The last three boxes are Random Walk of second order (RW2) models define using $U = 1, 2$ and 6 in the Penalised Complexity priors. The colours for the CRS, BS, TPRS, $U = 1$, $U = 2$, and $U = 3$ models are orange, sky blue, light green, yellow, blue, and light red, respectively

Figure 3 shows the results from the simulation study as a range of boxplots. The top-to-bottom rows are for the assessment criteria IS and 95% (uncertainty), width and (empirical) coverage. The left-to-right columns are for whether the predicted values are either estimates or forecasts. The boxplots of the MAE and MSE have no discernible differences for all six models (even when considering transformations of the *y*-axis). Consequently, we omit these from the Manuscript and include them in the Supplementary Material.

First, we considered the results for estimation. For all scores, there is minor difference within each model for the different specifications. Therefore, the results are robust to how the penalised spline and the RW2 are specified. For the IS, the RW2 models noticeably outperformed the penalised splines, with the Interquartile Range (IQR) of the boxplot for the RW2 models being below those of the penalised spline. When considering the widths, the RW2 models were larger than those of the penalised spline, and the coverage for the RW2 models was slightly larger. As the IS is a balance between penalising too large intervals and rewarding coverage, the RW2 models better IS score indicates that the penalised spline uncertainty intervals are too narrow.

Now, we considered the results for prediction. For all scores, the results are robust to the way each model is specified. The IS for the RW2 models are clearly lower than those of the penalised splines, the widths for the RW2 models are larger than those for the penalised splines, and the coverage is greater. When comparing the difference between the boxplots for the penalised splines and the RW2 in the estimation and forecasting results, the difference is larger in the forecasts than in the estimates. Therefore, the narrow widths of the penalised splines become more detrimental to the model performance, with respect to the distributional scores, as forecasts are subject to much more uncertainty than estimates, and the further forward the forecasts, the more uncertainty there is. The differences in the IS, width, and coverage are due to the way each measure quantifies uncertainty and defines the uncertainty interval.

### Using real-world data

To compliment the results of the simulated study, we compared the predictive results from a spline and random process using the real-world data. Based on the results of the simulation study, the outcomes were robust to the specification of the model, i.e., the type of spline basis and the prior specification. Therefore, for the alcohol and self-harm-related deaths examples, we used the TPRS spline basis, the mgcv default, and $U = 1$, the r-inla recommendation, to specify the penalised spline and RW2 models, respectively. For the alcohol and self-harm-related deaths datasets, we used all ages in the years 2006 to 2017 to fit the models and assess the models’ estimation (in-sample predictions) when compared to the real data. Using the models fit to the years 2006 to 2017, we forecasted the years 2018 to 2021 and used this window to assess the models forecasts when compared to the real data. Similar to the simulation study, we fit the models using a Poisson likelihood (presented in the Manuscript) and a negative Binomial likelihood (presented in the Supplementary Material) as a sensitivity analysis.

Table 1 shows the IS, width, and coverage (distributional scores) for both outcomes and each model partitioned into estimation and forecasting predictions. For both estimation and forecasting on both outcomes, the RW2 model always has the lower IS score, suggesting a better-performing model. Considering both the width and coverage. Whilst the RW2 has a larger width, the increased coverage due to this means the penalisation in the IS for the ‘true’ data being outside the interval is less than in the penalised spline model. Therefore, the RW2 models better distribution-based score is, in-part, due to the penalised spline producing too narrow intervals and not capturing the variation between the points as well. The outperformance of the RW2 model in comparison to the penalised spline model is more pronounced when considering forecasting.

**Table 1:** Model scores for the alcohol and self-harm related death data

Dataset	Model Type	Estimation $\left( \varvec{\times 10}^{\varvec{-2}}\right)$			Forecasting $\left( \varvec{\times 10}^{\varvec{-2}}\right)$		
		Interval Score	Width	95% Coverage	Interval Score	Width	95% Coverage
Alcohol	Spline	323.93	23.77	47.92	167.04	48.74	66.67
	RW2	281.96	26.56	56.25	110.71	93.69	91.67
Self-harm	Spline	95.90	9.57	50.00	108.54	18.16	58.33
	RW2	78.51	10.82	59.03	71.30	34.42	83.33

**Fig. 4:**
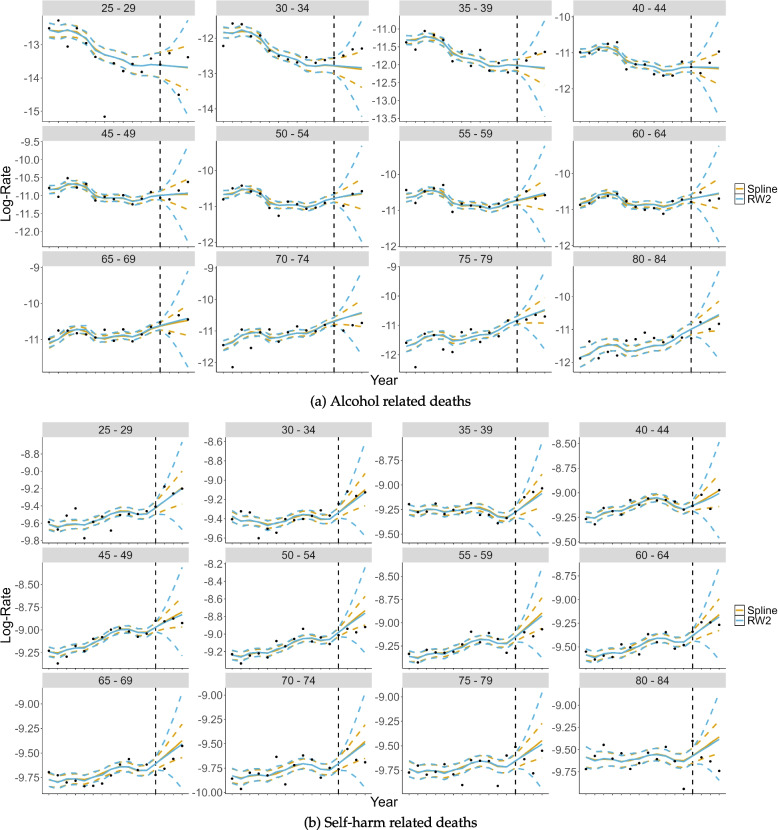
Estimated and predicted values of $\eta _{ap}$ for the spline and Random Walk of second order (RW2) models for alcohol (**a**) and self-harm (**b**) related suicides. In each subfigure, the facets are for each of the age groups increasing from left-to-right and top-to-bottom. The *y*-axis is the log rate and the *x*-axis is the year. The orange and sky blues solid lines are the fitted valued for the spline and RW2 models, respectively. The dashed lines are their associated uncertainty levels. The black dots are the true values, and the vertical black dotted line is where the estimation stops, and prediction begins

To further demonstrate why the RW2 model produced a better IS for both estimation and prediction than the penalised spline model, we presented the model estimates and forecasts against the real data for both outcomes in Fig. 4. In Fig. 4, the estimated values (solid lines) for the penalised regression spline (orange) and RW2 (sky blue) models, along with their associated lower and upper uncertainty intervals (dashed lines), are shown for each age group for alcohol (Fig. 4a) and self-harm (Fig. 4b) related deaths. We superimposed the real data (black dots) over the predictions. The better IS (a larger uncertainty with better coverage) for the RW2 models is clear when one of the black dots falls within the sky blue dashed lines, but outside the orange dashed lines. This is clearer to see when considering the predicted years (after the vertical black dashed line). For example, the last data point in facet 40-44 for alcohol-related deaths, Fig. 4a. This point falls within the RW2 uncertainty interval, but not the penalised spline’s uncertainty interval. Consequently, this would contribute to a better IS for the RW2 in comparison to the penalised spline. There are multiple more events such as this that contributed to the RW2’s overall better performance in terms of the IS.

## Discussion

In this article, we discuss the use of splines and random processes for making smooth predictions in the context of APC modelling. In the context of APC models, we discuss the theoretical link between model fitting via splines and random processes and then proceed to demonstrate in-sample and out-of-sample predictions for simulated and real data examples. Through both a theoretical and data driven links, we showed that model fitting and predictions using spline models and random process models are comparable in the context of APC models.

For both the simulated and real data examples, we assessed model performance using a range of scores, which we partitioned into scores for estimation (in-sample prediction) and forecasting (out-of-sample prediction). APC models are often used for predictive purposes, as forecasts for the burden of future health concerns are an important goal for many policymakers. When considering the point estimates only, the use of penalised splines and RW2 models is interchangeable. This is shown by the MAE and MSE from the simulation study having little-to-no difference at all. Furthermore, a similar conclusion can be made when considering the RW2 versus penalised spline plot line in the alcohol and self-harm-related deaths example. However, when one wishes to include uncertainty in the results, the results become different. The narrower confidence intervals of the spline approach are reflected in a model that does not capture variation in the data as well as a model fit using RW2 models in a Bayesian paradigm. The larger uncertainty of the results from a Bayesian paradigm compared to those of a frequentist paradigm have been noted previously in the context of APC modelling [61, 62]. The ‘Interval Score’ of Gneiting and Raftery [59], which defines a score that balances the width of the uncertainty interval and whether or not the observation falls within, highlights this. The inclusion of uncertainty in estimates is vital for policymakers as it allows them to base any future policy on the worst, middle, and best-case scenarios.

While the two methods share a common theoretical underpinning, the differences in our results can be attributed to how each method approaches smoothing in their respective software. For splines in mgcv, the smoothing parameter is estimated from the data using cross-validation. For random processes in r-inla, the smoothing parameter is defined *a priori* and is updated from the data. This needs to be done carefully as Bayesian methods can be prone to over/under-smoothing for a poor choice of prior. Given the similarities between the methods, if a researcher naively chooses one implementation over the other, our results show they should not worry about the predictions producing vastly different results. However, if the researcher were to make a nuanced choice to better account for uncertainty, they would choose to use random processes (implemented via the Bayesian paradigm).

Whilst predictions using an APC model, especially those fit in a Bayesian paradigm, do not suffer the identification problem like when estimating, predictions from identifiable models are considered more appropriate [63–65]. However, if a researcher wishes to avoid APC models and their identification issues, there are alternatives to the APC model that can be used. To avoid the co-linearity between the three temporal effects, an age-period interaction model or a model where the cohort is replaced with a proxy, as discussed by Clayton and Schifflers [66]. Both methods aim to replace the cohort with an equivalent term that is not linearly dependent on age and period. However, as we focus on identifiable APC models, we did not consider these methods here. Another class of models commonly used for mortality modelling is the Lee-Carter model [67]. We expect that our findings also extend to smoothing within this model, however, this is left for future work.

In conclusion, APC models are widely used tools for predicting health and demographic data, within which smoothing is a vital piece of the puzzle. Smooth predictions for APC models are implemented using two main schools: frequentist (penalised splines) and Bayesian (random processes) methods. While these two methods are interchangeable and produce equivalent results, we have shown that if a researcher wants to better capture the uncertainty of their data and provide a more complete set of predictions, then Bayesian models can prove beneficial. With software such as r-inla, model fitting via random processes has become increasingly more accessible, with the need for specialist knowledge reducing thanks to intuitive and reliable default choices within the software.

## Supplementary Information

Supplementary Material 1. We have provided all the relevant code and data in the following GitHub repository https://github.com/connorgascoigne/bayesian-splines. For the alcohol and self-harm related deaths illustration, we provide the code to run the analysis as well as the data we used, which fall under a UK Open Government License (https://www.nomisweb.co.uk/home/copyright.asp).

## Data Availability

We have provided all the relevant code and data in the following GitHub repository https://github.com/connorgascoigne/bayesian-splines. For the alcohol and self-harm related deaths illustration, we provide the code to run the analysis as well as the data we used, which fall under a UK Open Government License (https://www.nomisweb.co.uk/home/copyright.asp).
